# Cytological Observation and RNA-Seq Analyses Reveal *miR9564* and Its Target Associated with Pollen Sterility in Autotetraploid Rice

**DOI:** 10.3390/plants13111461

**Published:** 2024-05-24

**Authors:** Zijun Lu, Weicong Huang, Lianjun Zhu, Guobin Liang, Yu Huang, Jinwen Wu, Rou Chen, Xiang Li, Xiangdong Liu

**Affiliations:** 1 State Key Laboratory for Conservation and Utilization of Subtropical Agro-Bioresources, Guangdong Laboratory for Lingnan Modern Agriculture, South China Agricultural University, Guangzhou 510642, China; luzj@scau.edu.cn (Z.L.); huangwc@stu.scau.edu.cn (W.H.); zhulianjun@stu.scau.edu.cn (L.Z.); liangguobin@stu.scau.edu.cn (G.L.); hy@stu.scau.edu.cn (Y.H.); jwwu@scau.edu.cn (J.W.); chenrou187@stu.scau.edu.cn (R.C.); 2 Guangdong Provincial Key Laboratory of Utilization and Conservation of Food and Medicinal Resources in Northern Region, Shaoguan University, Shaoguan 512005, China; 3 Guangdong Provincial Key Laboratory of Plant Molecular Breeding, South China Agricultural University, Guangzhou 510642, China; 4 Guangdong Base Bank for Lingnan Rice Germplasm Resources, College of Agriculture, South China Agricultural University, Guangzhou 510642, China

**Keywords:** polyploid rice, autotetraploid, pollen sterility, meiosis, miRNA

## Abstract

Understanding the regulation of autotetraploid sterility is essential for harnessing the strong advantages in genomic buffer capacity, biodiversity, and heterosis of autotetraploid rice. miRNAs play crucial roles in fertility regulation, yet information about their reproductive roles and target genes in tetraploid rice remains limited. Here, we used three tetraploid lines, H1 (fertile), HF (fertile), and LF (sterile), to investigate cytological features and identify factors associated with autotetraploid sterility. LF showed abnormal meiosis, resulting in low pollen fertility and viability, ultimately leading to scarce fertilization and a low-seed setting compared to H1 and HF. RNA-seq revealed 30 miRNA-candidate target pairs related to autotetraploid pollen sterility. These pairs showed opposite expression patterns, with differential expression between fertile lines (H1 and HF) and the sterile line (LF). qRT-PCR confirmed that *miR9564*, *miR528*, and *miR27874* were highly expressed in the anthers of H1 and HF but not in LF, while opposite results were obtained in their targets (*ARPS*, *M2T*, and *OsRPC53*). Haplotype and expression pattern analyses revealed that *ARPS* was specifically expressed in lines with the same haplotype of *MIR9564* (the precursor of *miR9564*) as LF. Furthermore, the Dual-GFP assay verified that *miR9564* inhibited the fluorescence signal of ARPS-GFP. The over-expression of *ARPS* significantly decreased the seed setting rate (59.10%) and pollen fertility (50.44%) of neo-tetraploid rice, suggesting that *ARPS* plays important roles in autotetraploid pollen sterility. This study provides insights into the cytological characteristic and miRNA expression profiles of tetraploid lines with different fertility, shedding light on the role of miRNAs in polyploid rice.

## 1. Introduction

Regardless of autopolyploidy and allopolyploidy, polyploid individuals have substantial advantages in stress resistance, biosynthesis, genomic buffering capacity, biodiversity, and heterosis [1,2,3]. However, the widespread complex reproductive defects in autotetraploid plants limit their application [4,5,6,7]. Autotetraploid rice, resulting from doubling its diploid counterpart, represents a promising breeding germplasm, which displays high salt and heavy metal ion resistance [8,9,10,11]. Partial pollen sterility stands out as a major factor contributing to a low-seed set of autotetraploid rice. Within this context of pollen sterility, abundant abnormalities had been observed in the meiotic anthers of autotetraploid rice, including abnormal chromosome behavior and tapetum development [12,13,14]. Chinese scientists dedicated at least 20 years in repeated selfing and selection to overcome polyploid infertility, resulting in the successful breeding of fertile tetraploid rice, such as PMeS polyploid rice and neo-tetraploid rice (NTR, with 80% seed setting) [15,16,17,18]. Notably, NTR lines have shown the ability to overcome polyploidization sterility when crossed with typical autotetraploid rice exhibiting low fertility [16]. These fertile tetraploid rice germplasms have produced a valuable opportunity to identify genes associated with autotetraploid infertility. Our previous studies had reported 15 NTR lines to assess their yield traits, reproduction, and gene expression [16,17,18]. Relative to diploid counterparts and NTR lines, great changes have been detected in expression levels of genes, microRNAs (miRNAs), and long non-coding RNAs during embryo sac development in autotetraploid rice, such as meiotic genes [12,13,14,19].

miRNA is a special non-coding RNA that targets specific areas of mRNA to suppress gene expression by a direct cleaving of mRNAs by miRNAs or inhibiting the translation of the target mRNA to participate in numerous crucial processes [20]. For instance, *miR528* regulates pollen intine formation by targeting the uclacyanin gene *OsUCL23* to impact flavonoid metabolism [21], modulates flowering time by targeting *OsRFI2* [22], and regulates *OsSPL9* to affect the antiviral response [23]. *miR2118* regulates reproductive tissue development in rice [24]. Additionally, *miR167d*, *miR398b*, *miR7695*, and *miR1873* are associated with blast resistance [25,26,27,28]. *miR156* defines ideal plant architecture by targeting *OsSPL14* and regulates seed dormancy through the gibberellin pathway [29,30]. Recently, the regulatory network of *OsPIL15*-*miR530*-*OsPL3* and *miR1432*-*OsACOT* was found to be related to rice grain yield [31,32].

From diploidy to autotetraploidy, the expression patterns of miRNA dynamically changed in the anther and ovary, indicating the important roles of miRNAs in autotetraploid reproduction [12,13,16]. There were 172 miRNAs differentially expressed in the meiosis anther of autotetraploid rice, including 57 miRNAs specifically expressed in autotetraploid rice [12]. Furthermore, 321 and 368 miRNAs showed differential expression during anther and embryo sac development in autotetraploid rice, respectively [13]. In addition, 122 miRNAs were differentially expressed in the meiotic anther of the neo-tetraploid line H3 compared to autotetraploidy T452 [16]. However, the functional verification about miRNAs and their negative targets in the reproductive regulation of tetraploid rice is still limited.

In this study, two neo-tetraploid lines (H1 and HF) and one low-fertility tetraploid line (LF) were utilized for cytological observation, miRNA-seq, and RNA-seq to identify common differentially expressed miRNAs and their negative targets relative to tetraploid reproduction. One key candidate target was further over-expressed in neo-tetraploid rice. As expected, the transgenic materials exhibited a low-seed set and low pollen fertility. These findings contributed to enrich our understanding of pollen development in autotetraploid rice.

## 2. Results

### 2.1. The Low-Fertility Tetraploid Line (LF) Exhibited Defects in Pollen Development and Fertilization

**Figure 1:**
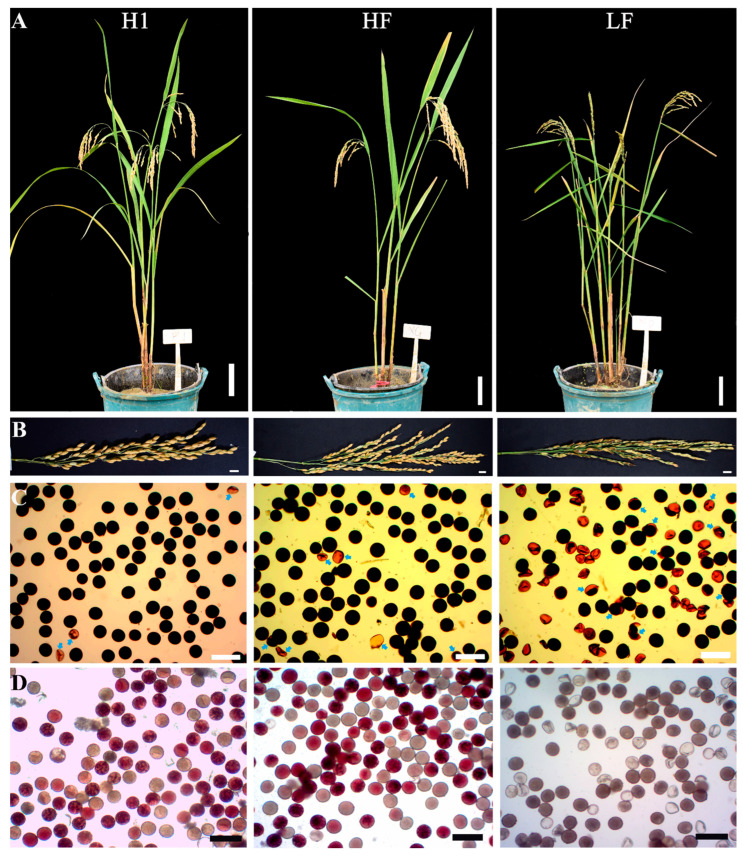
Morphological and cytological observations of H1, HF, and LF. (**A**,**B**) Plant and panicle types of H1, HF, and LF. (**C**) Pollen grains stained with 1% I_2_/KI solution. (**D**) Pollen grains stained with 1% TTC solution. LF exhibited a high frequency of aborted pollen grains (arrows) and low viability. Bars = 10 cm (**A**), 1 cm (**B**), 100 µm (**C**,**D**).

**Figure 2:**
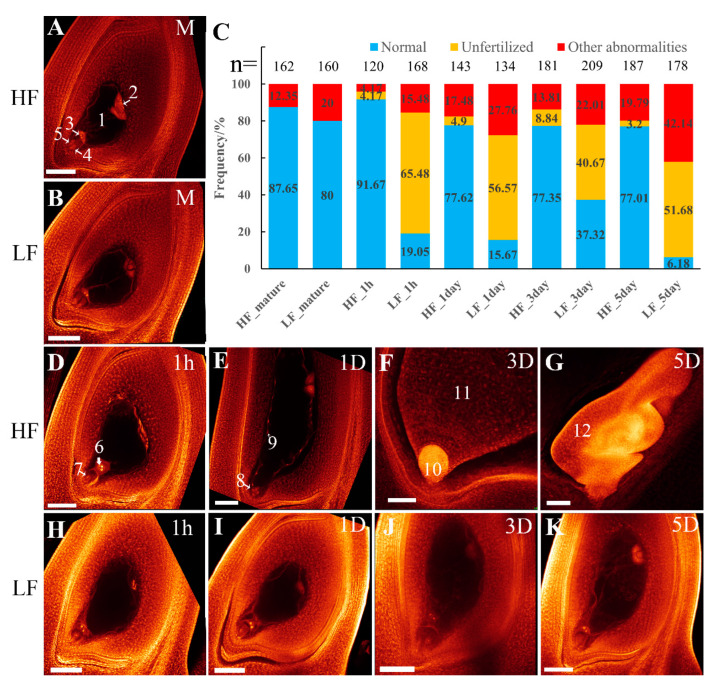
Double fertilization and embryogenesis in LF and HF. (**A**,**B**) Mature embryo sac in HF and LF. (**C**) Frequency distribution of embryo sac development and double fertilization; *n*, total number of ovules. (**D**–**G**) Normal ovaries in HF at 1 h after flowering (1 h), 1 day after flowering (DAF) (1D), 3 DAF (3D), and 5 DAF (5D). (**H**–**K**) Infertile ovaries in LF. 1, central cell; 2, antipodal cells; 3, polar nuclei; 4, synergid; 5, egg cell; 6, sperm nucleus approaches polar nuclei at 1 h; 7, sperm nucleus enters egg cell at 1 h; 8, globular embryoid at 1 DAF; 9, free endosperm nucleus at 1 DAF; 10, pear-shaped embryoid at 3 DAF; 11, endosperm at 3 DAF; 12, embryo at 5 DAF. M, mature; h, hour; D, days after flowering. Bars, 100 µm.

An F_2_ population (1409 individuals) was constructed from the combination of fertile Huaduo1 (H1, neo-tetraploid rice) and sterile Linglun-4x (autotetraploid rice) in 2012. Two groups of plants were selected: group 1 (113 individuals, seed setting ≥ 88%) and group 2 (75 individuals, seed setting ≤ 40%). From F_3_ to F_11_, group 1 maintained fertile selfing progenies (seed setting rate ≥ 70%), while group 2 maintained sterile progenies (seed setting rate ≤ 20%). From 2017 (F_11_), a fertile line designated as High-Fertility Tetraploidy (HF) was selected from group 1, while another sterile line designated as Low-Fertility Tetraploidy (LF) was selected from group 2 (Figure S1A,B and Figure 1A). The plant height (16.48–18.19% reduction), panicle length (15.16–17.76% reduction), and grain number per panicle (17.02–28.60% reduction) of LF were significantly lower than that of HF (Figure S1C–E). The successive generations of HF and LF displayed stable fertility and sterility, respectively. The seed setting of LF (5.17%) was significantly lower than that of HF (71.39%) and H1 (74.38%) (Table 1, Figure 1A,B). A high-seed setting was observed in hybrids of T428 × H1 (86.72%), LF × HF (75.30%), and LF × H1 (79.43%) (Figure S2), suggesting the presence of dominance gene action for high fertility. The pollen grains developed normally in HF (90.31%) and H1 (97.10%), whereas a large number of aborted pollen grains were found in LF (only 26.02% normal pollen grains) (Table 1, Figure 1C). The 2,3,5-Triphenyl-2H-tetrazolium chloride (TTC) solution was applied to detect pollen viability, and the results showed that the pollen viability of H1, HF, and LF was 46.93%, 65.75%, and 17.95%, respectively (Table 1, Figure 1D). Additionally, whole-mount eosin B-staining confocal laser scanning microscopy (WE-CLSM) observations of mature embryo sacs revealed that HF and LF had a polygonum-type embryo sac (Figure 2A,B), with embryo sac fertility of 87.65% for HF and 80.00% for LF, respectively (Figure 2C).

Moreover, the embryogenesis and endosperm development of HF and LF were also observed using WE-CLSM at 1 h after flowering, 1 day after flowering (DAF), 3 DAF, and 5 DAF. In HF, 91.67%, 77.62%, 77.35%, and 77.01% samples were able to be fertilized at 1 h, 1 DAF, 3 DAF, and 5 DAF, respectively, while only 19.05%, 15.67%, 37.32%, and 6.18% of observed samples were fertilized in LF (Figure 2C–G). Most of the samples in LF were still unfertilized and retained the mature embryo-sac-like morphology (Figure 2H–K) or exhibited other abnormalities (Figure S3). These results indicate that LF displayed defects during pollen development, double fertilization, and embryogenesis.

### 2.2. LF Showed Severe Abnormal Male Meiotic Process

**Figure 3:**
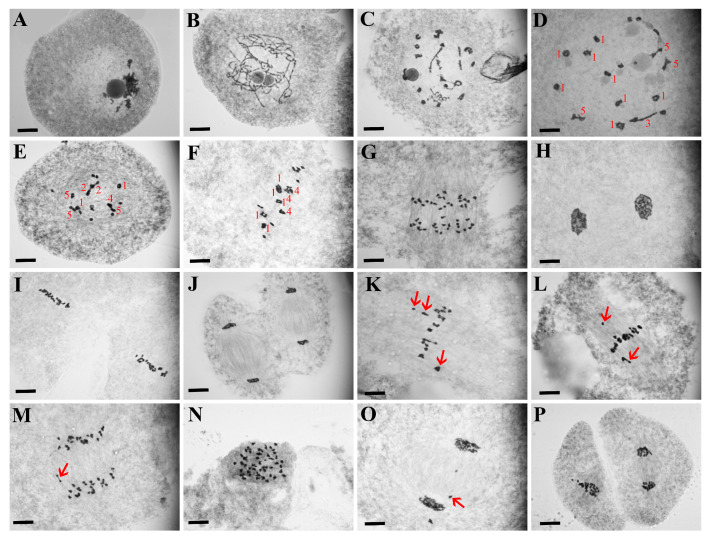
Pollen mother cell chromosome behavior during meiosis in LF (low-fertility tetraploid line). (**A**) Zygotene; (**B**) Pachytene; (**C**) Diplotene; (**D**) Diakinesis; (**E**) Pre-metaphase I; (**F**) Metaphase I; (**G**) Anaphase I; (**H**) Telophase I; (**I**) Metaphase II; (**J**) Anaphase II; (**K**,**L**) Chromosome dragging at metaphase I (arrows); (**M**) Chromosome lagging at anaphase I (arrows); (**N**) Unordered chromosomes at anaphase I; (**O**) Micronuclei at telophase I (arrows); (**P**) Asynchrony of the chromosome during meiosis II. Red number indicates different-type quadrivalents: 1, ring-shaped; 2, chain-shaped; 3, frying pan-shaped; 4, OK-shaped; 5, X-shaped. Bars, 10 μm.

**Figure 4:**
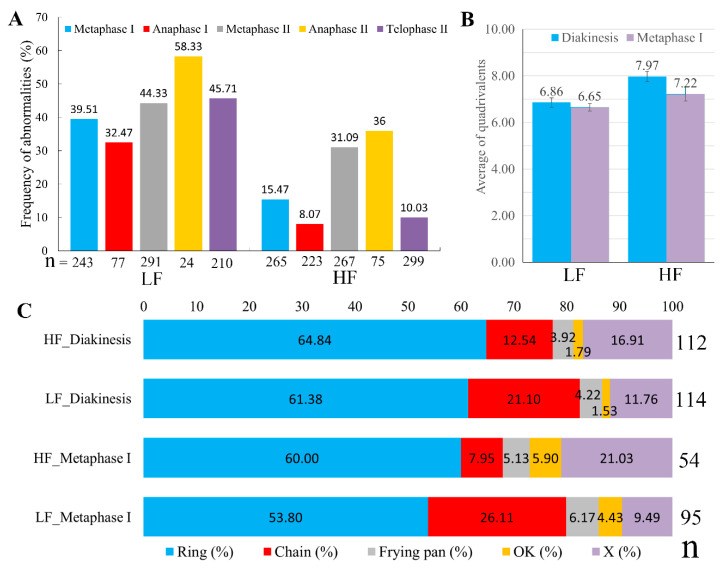
Frequency of abnormal chromosome behavior and quadrivalent distribution in meiotic pollen mother cells. (**A**) Frequency of abnormalities in LF and HF during different stages of meiosis. (**B**) Average of quadrivalents in LF and HF at diakinesis and metaphase I. (**C**) Frequency of different types of quadrivalents at diakinesis and metaphase I. “Ring”, “Chain”, “Frying pan”, “OK”, and “X” indicate different-type quadrivalents shown in Figure 3D–F. *n*, number of pollen mother cells observed.

Similar to diploid rice, HF underwent meiosis with fewer abnormalities. However, more abnormal chromosome behaviors were observed in meiotic pollen mother cells (PMCs) of LF, including chromosome dragging at metaphase I and metaphase II, chromosome lagging at anaphase I and anaphase II, chromosome behavior disorder, micronuclei at telophase I and telophase II, abnormal cell shape and asynchrony of the chromosome during meiosis II, and an abnormal tetrad (Figure 3). High frequencies of chromosome behavior abnormalities were observed since the early stage of meiosis in LF, which were significantly higher than HF at all observed stages (Figure 4A). Abnormal chromosome configurations were found in both LF and HF, such as univalents, trivalents, and particularly quadrivalents. The number of quadrivalents per cell was lower in LF (6.86) compared to HF at diakinesis (7.97, Figure 4B). A ring shape was the most frequent configuration of quadrivalents in both HF and LF. HF had a higher frequency of ring-shape (64.28%) and X-shape (16.91%) quadrivalents than LF (57.93%, 11.76%, respectively), while LF had a higher frequency of chain-shaped quadrivalents (21.10%) than HF (12.54%, Figure 4C). A similar result was observed during metaphase I (Figure 4B,C).

### 2.3. Comparative miRNA Expression Profiles in Meiotic Anthers of H1, HF, and LF

To investigate the differences in miRNA expression during anther development among tetraploid lines with opposite fertility, three small RNA libraries from meiotic anthers of H1, HF, and LF were sequenced by using Illumina Solexa high-throughput sequencing technology. A total of 38,569,001, 39,942,383, and 45,913,426 raw reads were generated, yielding 28,129,531 (72.93%), 27,818,269 (69.65%), and 27,648,526 (60.22%) valid reads from H1, HF, and LF, respectively (Table S1). A significant correlation was observed among three biological replicates of each library with a correlation coefficient exceeding 0.95 (Figure S4).

**Figure 5:**
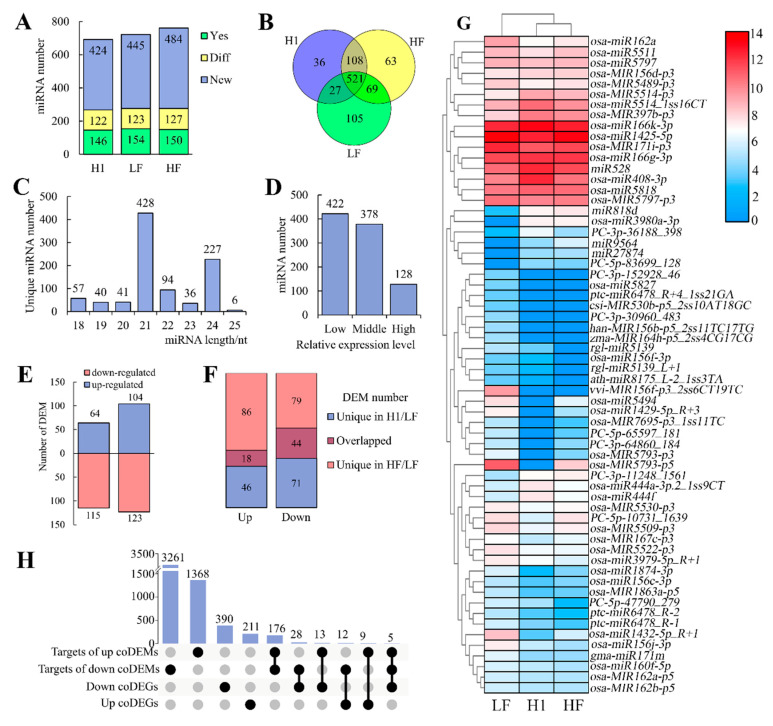
Identification of miRNAs from meiotic anthers of H1, HF, and LF. (**A**) Number of miRNAs detected in H1, HF, and LF. Yes, miRNAs detected in miRBase. Diff, miRNAs detected in miRBase with differing sequence. New, miRNAs not present in miRBase. (**B**) Venn diagram analysis of miRNAs among H1, HF, and LF. (**C**) Length distribution of unique miRNAs. (**D**) Number of miRNAs with different expression levels, categorized as High, Middle, and Low. (**E**) Number of differentially expressed miRNAs (DEMs) in H1 and HF; (**F**) identification of coDEMs between H1 and HF; (**G**) hierarchical cluster analysis among coDEMs; (**H**) upset plot analysis of coDEM targets and coDEGs to identify negative regulatory groups. DEGs, differentially expressed genes.

A total of 929 miRNAs were detected across the three libraries (Table S2). A total of 692 miRNAs were detected in H1, comprising 268 known and 424 novel miRNAs. Among these 268 known miRNAs, 146 miRNAs showed an identical sequence compared to miRBase (labeled as “Yes”) and others had a different sequence (labeled as “Diff”). In HF, 761 miRNAs were detected, consisting of 277 known (150 Yes and 127 Diff) and 484 novel miRNAs. Similarly, 722 miRNAs were detected in LF with 277 known (154 Yes and 123 Diff) and 445 novel miRNAs (Figure 5A). A total of 521 miRNAs were shared in all three libraries, while 36, 63, and 105 miRNAs were specifically detected in H1, HF, and LF, respectively (Figure 5B). The length distribution of mature miRNAs was primarily enriched in 21 nt and 24 nt (Figure 5C). Among these miRNAs, 128 showed high expression levels (FPKM > 260.81), 422 displayed low expression levels (FPKM < 10.00), and the remaining 378 were considered as moderately expressed. The 422 low-expressed miRNAs were subsequently excluded from the further analysis (Figure 5D).

In comparison to LF, 179 differentially expressed miRNAs (DEMs) were identified in H1, including 64 up-regulated (up) and 115 down-regulated (down) DEMs (Table S3). Among them, 24 up- and 33 down-DEMs displayed high expression levels. HF showed 227 DEMs, with 104 up- and 123 down-DEMs (Figure 5E, Table S4). The Venn diagram analysis revealed that 18 DEMs (5.80%) were co-up-regulated (cuDEM), while 44 DEMs (14.10%) were co-down-regulated (cdDEM) in HF and H1 (Figure 5F, Table S5). These 62 coDEMs were considered as candidate miRNAs related to autotetraploid pollen fertility (Figure 5G).

To gain further insight into the functions of coDEMs, we conducted an analysis of their potential targets. In total, 5944 mRNAs transcribed by 4866 genes were annotated as potential targets of 60 coDEMs. Gene Ontology (GO) analyses of 3476 predicted target genes of 44 cdDEMs revealed significant enrichment in various processes including the protein modification process (GO: 0006464), regulation of gene expression and epigenetics (GO: 0040029), signal transduction (GO: 0007165), pollen–pistil interaction (GO: 0009875), cell differentiation (GO: 0030154), post-embryonic development (GO: 0009791), anatomical structure morphogenesis (GO: 0009653), plasma membrane (GO: 0005886), kinase activity (GO: 0016301), and nucleotide binding (GO: 0000166) (Figure S5). Most of the enriched GO items were associated with reproductive development, suggesting that these coDEMs might play important roles during reproductive development in tetraploid rice.

### 2.4. Identification of Negative Regulative miRNA-Target Pairs during Meiosis in Neo-Tetraploid Rice

**Figure 6:**
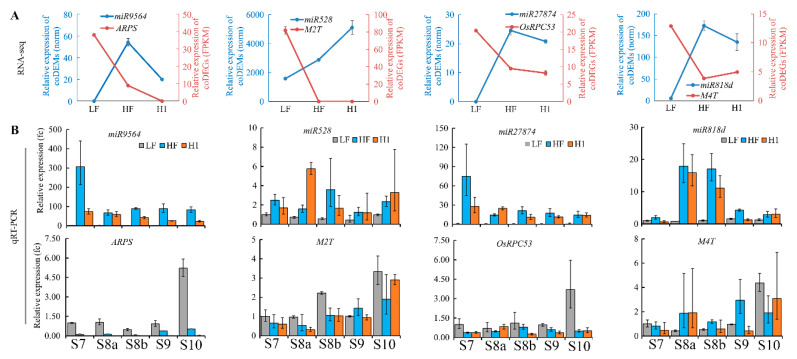
Expression analyses of four key coDEMs and coDEGs in developing anthers. Expression levels of *miR9564*-*ARPS*, *miR528*-*M1T*, *miR27874*-*OsRPC53*, and *miR818d*-*M4T* were analyzed using RNA-seq data (**A**) and qRT-PCR (**B**) in H1, HF, and LF anthers. Fc, fold change relative to sample with lowest expression. *Ubiquitin* was used as internal control gene. S7 to S10 represent anther stages. Error bars indicate standard error (SE) with *n* = 3.

Previously, the same RNA samples were used for RNA-seq analyses, which identified 668 common differentially expressed genes (coDEGs) in meiotic anthers of HF and H1 relative to LF, comprising 232 up- (cuDEGs) and 436 down-regulated coDEGs (cdDEGs) (Table S6) [33]. The Venn diagram analysis of coDEGs and coDEMs–targets identified 30 miRNA-target pairs demonstrating negative regulatory relationships between miRNAs and their targets (Figure 5H, Table S7). Among them, four cuDEGs, *LOC_Os11g17290* (named as *ARPS* here, predicted target of *miR9564*), *LOC_Os02g06760* (named as *M2T* here, predicted target of *miR528*), *LOC_Os04g32350* (*OsRPC53*, predicted target of *miR27874*), and *LOC_Os06g38210* (named as *M4T* here, predicted target of *miR818d*), showed high expression levels (FPKM > 10) in meiotic anthers and significant differences in the expression level between HF/H1 and LF (Figure 6A).

qRT-PCR was performed to verify the expression levels of the four aforementioned coDEM–DEG pairs during anther development of H1, HF, and LF. *miR9564* showed up-regulated expression in developmental stage 7 to 10 (S7–S10) anthers of HF and H1 compared to LF, while its target *ARPS* showed down-regulated expression during the same stage. Similarly, *miR528* and *miR27874* displayed up-regulated expression in S7–S10 anthers of HF and H1, while their targets *M2T* and *M3T* showed down-regulated expression during the same stage. Another cuDEM, *miR818d*, was confirmed with up-regulated expression in S8a–S8b anthers of HF and H1, but its potential target *M4T* was also up-regulated in S8a anthers. *M4T1* encoded an expressed protein without known function and was mainly highly expressed in vegetative organs. Opposite expression patterns were found in *miR9564*-*ARPS*, *miR528*-*M2T*, and *miR27874-M3T*, but not in *miR818d-M4T* (Figure 6B). Because of its most substantial expression differences, the *miR9564-ARPS* couple was selected as autotetraploid rice pollen sterility candidates for further verification.

### 2.5. miR9564 Could Negatively Regulate the Expression Level of ARPS

**Figure 7:**
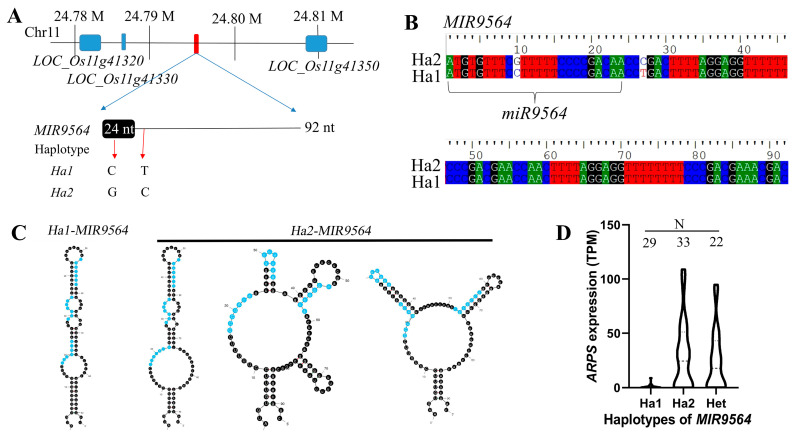
The relationship between *MIR9564* haplotypes and *ARPS* expression levels. (**A**,**B**) Information about the precursor of *miR9564* (*MIR9564*). Ha1 and Ha2 indicate two haplotypes of *MIR9564*. (**C**) Predicted RNA secondary structures of *Ha1-MIR9564* and *Ha2-MIR9564*; (**D**) expression levels of *ARPS* in plants with different *MIR9564* haplotypes. Het indicates the heterozygote of Ha1 and Ha2. N, indicated numbers of RNA-seq samples for expression analyses of *ARPS*.

*miR9564* was 24 nt long and its precursor *MIR9564* was 92 nt long on chromosome 11. A re-sequencing analysis revealed different haplotypes of *MIR9564* between HF-H1 and LF. The haplotype in HF and H1 (labeled as Ha1) contained two SNPs, including the 10th G:C and the 27th C:T, while the haplotype in LF (labeled as Ha2) was the same as the reference genome (Figure 7A,B). The mRNAs of *Ha2-MIR9564* were predicted to form three RNA secondary structures different from that of *Ha1-MIR9564* (Figure 7C). Interestingly, previously finished RNA-seq and re-sequencing datasets revealed that 33 samples with *Ha2-MIR9564* and 22 samples with heterozygous *MIR9564* displayed a high expression level of *ARPS*, but not in 29 samples with *Ha1-MIR9564* (Figure 7D).

**Figure 8:**
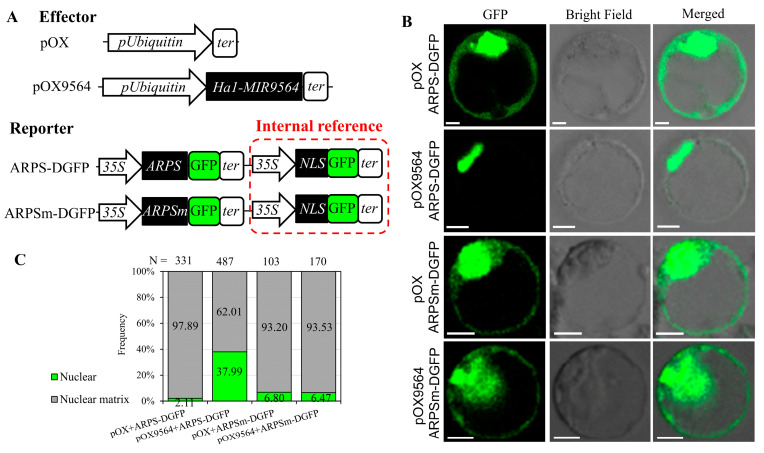
Dual-GFP assay for negative regulation between *MIR9564* and *ARPS*. (**A**) Schematic diagrams illustrating information about effectors and reporters. NLS, nuclear localization signal; DG, Dual-GFP. (**B**) Subcellular localization of Dual-GFP signal for testing *MIR9564* regulation on ARPS-GFP activity. (**C**) Frequency of protoplasts with different types of GFP signals. N, number of observed protoplasts.

To verify if the expression level of *ARPS* is repressed by *miR9564*, a Dual-GFP assay was further performed, which used a nuclear-localized GFP signal as the internal control (Figure S6). In the control, strong ARPS-GFP fluorescence was distributed in both the nucleus and cytoplasm of protoplasts. While co-expressing the *Ubi::MIR9564* effecter (*pOX9564*) and APRS-DGFP reporter, APRS-GFP signals were repressed in the cytoplasm, and only nuclear GFP signals were observed. Once *ARPS* was mutated in the target site, the *pOX9564* effecter lost its ability to repress the fluorescence signal of ARPSm-GFP (Figure 8). Taken together, these results suggested that *miR9564* might negatively affect the expression level of *ARPS*.

### 2.6. Over-Expression of ARPS Reduced Pollen Fertility and Seed Set in Neo-Tetraploid Rice

**Figure 9:**
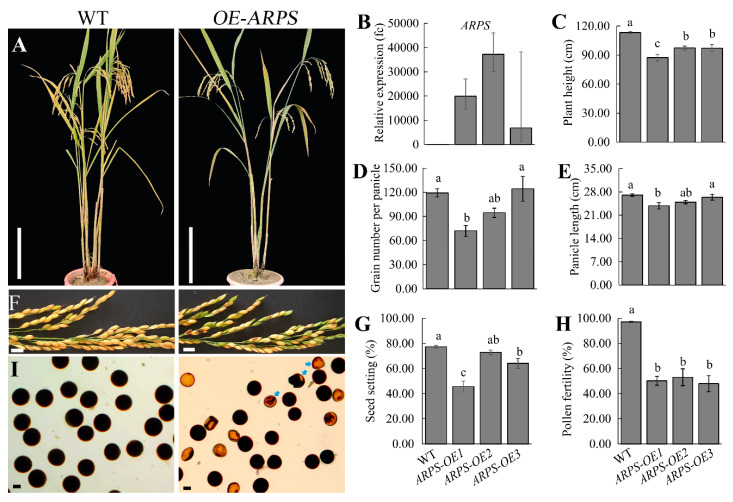
Morphological identification of *ARPS-OE*. (**A**) Plant morphology of WT (H1) and *ARPS-OE*. (**B**) Expression level of *ARPS* in WT and *ARPS-OE.* (**C**–**I**) Plant height (**C**), grain number per panicle (**D**), panicle length (**E**), mature panicles (**F**), seed setting rate (**G**), I_2_/KI-stained pollen fertility (**H**,**I**) of WT and *ARPS-OE*. Aborted pollen grains are marked by arrows. Bars = 20 cm (**A**), 1 cm (**F**), and 20 μm (**I**). Error bars indicate the SE with *n* ≥ 3. Significant differences are indicated by different lowercase letters (one-way ANOVA, least significant difference (LSD) test, *p* < 0.05).

To further understand the reproductive roles of *ARPS*, three over-expression lines of *ARPS* driven by the *Ubique* promoter (*ARPS-OE1*, *ARPS-OE2*, and *ARPS-OE3*) were constructed in the H1 background (Figure 9A). *ARPS* showed 6856.03~37238.58-fold up-regulation in obtained *ARPS-OE* lines (Figure 9B). The plant height (14.05–22.85% reduction), panicle length (2.47–11.95% reduction), and grain number per panicle (0.00–39.56% reduction) slightly reduced relative to WT plants (Figure 9C–E), which is consistent with the difference between HF and LF (Figure S1C–E). As expected, the seed setting rates of *ARPS-OE1* (45.68%) and *ARPS-OE3* (64.12%) were significantly lower than that of WT plants (77.27%) (Figure 9F,G). Moreover, abundant abortive pollen grains were found in *ARPS-OE* plants. The pollen fertility of *ARPS-OE* lines ranged from 48.00% to 53.03%, which was significantly lower than that of WT plants (97.10%) (Figure 9H,I). These results indicate that the up-regulation of *ARPS* expression in anthers would be detrimental to normal pollen development.

## 3. Discussion

In recent years, various factors affecting autotetraploidization sterility have been identified, including meiotic genes, miRNAs, long non-coding RNA, and DNA methylation sites [6,10,11,12,13,14,19,33,34]. Among these regulators, miRNAs with variable expression patterns have been proposed as an important factor for low fertility of autotetraploid rice. In comparison to Taichung65, 172 differentially expressed miRNAs were identified from anthers of autotetraploid rice Taichung65-4x [12]. Relative to 02428-2x, 321 and 368 miRNAs were differentially expressed in anthers and ovaries of autotetraploid rice 02428-4x, respectively [13]. Additionally, some differentially expressed miRNAs in the meiotic anther relative to the fertile neo-tetraploid lines were identified, such as *osa-miR408-3p* and *osa-miR528-5p* [16,34].

In this study, 62 coDEMs were identified from meiotic anthers of two neo-tetraploid lines to further enrich the understanding about miRNAs related to autotetraploid rice pollen sterility. Moreover, 30 miRNA-target pairs were highlighted because of their opposite expression patterns in both H1/LF and HF/LF comparative RNA-seq analyses. One of these coDEMs, *miR528*, has been previously reported to regulate pollen intine formation by targeting *OsUCL23* to influence flavonoid metabolism [21]. The STTM (Short Tandem Target Mimic) lines of *miR528* in neo-tetraploid rice exhibited a significant reduction in seed setting and pollen fertility [34]. Here, we identified the *OsINH2* gene as a novel candidate target of *miR528*, which functions in regulating pollen viability and grain numbers [35]. In both H1/LF and HF/LF, *miR528* was up-regulated during pollen development, while *OsINH2* showed down-regulation. The altered expression pattern of *miR528*-*OsINH2* from sterile autotetraploid rice to fertile neo-tetraploid rice suggests its potential importance in enhancing pollen fertility.

*OsRPC53* encodes a subunit of RNA polymerase C (III), which is required for the hybrid pollen sterility, grain length, and grain number in rice [36,37]. The mutation of *OsRPC53* caused pollen sterility in interspecific hybrid progeny of cultivated rice and Asian annual wild rice (*Oryza nivara*) [36]. In this study, a negative relationship in expression between *miR27874* and *OsRPC53* was identified and verified by qRT-PCR. *miR27874* showed high-level expression in developing anthers of H1 and HF, but not in LF, while its target, *OsRPC53*, maintained a high expression level in LF anthers. These results suggest that *miR27874*-*OsRPC53* might play important roles in regulating pollen fertility of neo-tetraploid rice.

In rice, *STS1* encodes an endoplasmic reticulum-localized protein with lipase activity, essential for tapetal degeneration and pollen wall formation [38]. In this study, the expression of *STS1* was up-regulated in both H1 and HF compared to LF, while its regulator miRNA, *osa-MIR5522-p3*, was down-regulated. These results suggested that the expression level of *STS1* may be insufficient for post-remodeled reproduction in autotetraploid rice, leading to partial pollen sterility. However, the down-regulated expression of *osa-MIR5522-p3* might facilitate the rescue of a higher expression level of *STS1* during pollen development in neo-tetraploid rice, contributing to its high pollen fertility.

*miR9564* was initially identified in the flowers of *Brassica rapa*, but its function remained unknown [39]. Here, we identified the homologous *miR9564* from the meiosis anther of H1 and HF, which displayed a high expression level in developing anthers of H1 and HF, but not in LF. Conversely, its predicted target (*ARPS*) maintained a high expression level only in LF anthers. Moreover, the Dual-GFP assay revealed that the expression of *MIR9564* could inhibit the fluorescence signals of ARPS-GFP, but not in mutant ARPS-GFP. These results indicate that *ARPS* is a target of *miR9564*. *ARPS* is a homologous gene of *AtULP1a* from *Arabidopsis*, which encodes an ESD4-like SUMO (small ubiquitin-related modifier) protein kinase 1 [40]. SUMO is a micromolecule with a similar structure to ubiquitin molecules and participates in protein modification after translation. It has been found that the SUMO genes regulate the nitrogen homeostasis, grain size, anther dehiscence, pollen fertility, and seed set in rice [41,42]. In this study, two haplotypes of *MIR9564* were identified, in which one haplotype related to H1 and HF exhibited a low expression level of *ARPS*. Furthermore, the over-expression of *ARPS* was detrimental to pollen fertility and seed setting in neo-tetraploid rice. These results suggested that the high expression of *miR9564* may suppress *ARPS* expression, preventing the partial autotetraploid pollen sterility caused by polyploidization in neo-tetraploid rice.

Taken together, our results unveil the regulatory roles of *miR528*-*OsINH2*, *miR27874*-*OsPRC53*, *miR5522-p3*-*STS1*, and *miR9564*-*ARPS* in autotetraploid pollen sterility, providing a unique perspective on the mechanisms underlying sterility in autotetraploid rice.

## 4. Materials and Methods

### 4.1. Plant Material

Anthers during meiosis of two fertile neo-tetraploid lines, Huaduo1 (H1) and High-Fertility Tetraploidy (HF), and one low-fertility tetraploid line (LF), were used for miRNAs’ analysis. HF and LF were sister lines derived from the progenies of Linglun-4x (infertile) × H1. H1 was the receptor for over-expression lines of *ARPS*.

### 4.2. Cytological Observation

Three florets from each of the three plants for each line were collected for evaluation on pollen fertility or viability. Pollen grains fixed in a Carnoy solution (ethanol/acetic acid = 3:1 *v*/*v*) for a minimum of 24 h were stained by a 1% iodine–potassium iodide solution (*w*/*v*) to evaluate pollen fertility. Pollen viability was evaluated with live pollen grains subjected to a 1% 2,3,5-Triphenyl-2H-tetrazolium chloride (TTC, *w*/*v*) solution at 31 °C. Subsequently, viable pollen grains turned red, while devitalized pollen grains retained their original color (Figure S7). Each sample was then photographed, obtaining five pictures from different perspectives under a Motic BA200 microscope, to count the pollen grains.

Florets were collected and fixed in the Carnoy solution for at least 24 h. Pollen mother cells were isolated and placed into a 1% acetocarmine solution for 3–5 min. Slides were briefly exposed to a flame for 5–7 s to induce thermal expansion and squashed to obtain separated meiotic chromosomes. Meiotic chromosomes were observed and photographed under a Motic BA200 microscope. The quantification of chromosome behaviors and chromosome configurations was performed as described in our previous study [18].

Whole-mount eosin B-staining confocal laser scanning microscopy (WE-CLSM) observations were performed to characterize embryo sac fertility, endosperm development, and embryogenesis in ovaries or developing seeds, as described in our previous study [33]. Those collected samples were fixed in an FAA solution (70% ethanol/acetic acid/methanal = 89:5:5, *v*/*v*), went through gradient rehydration (50%, 30%, 10%, and 0% ethanol, *v*/*v*), were stained by a 4% eosin B solution (*w*/*v*), were dehydrated by gradient ethanol (10%, 30%, 50%, 70%, 90%, 100% × 3, *v*/*v*), and were hyalinized via 50% (ethanol/methyl salicylate = 1:1, *v*/*v*) and pure methyl salicylate before observation under WE-CLSM. WE-CLSM was performed on Leica TCS SP2 under a 532 nm laser with an emission range from 545 to 638 nm.

### 4.3. miRNA Analysis

RNA samples were extended from anthers during meiosis, which were used for RNA-seq [33]. Small RNA libraries were constructed by Illumina’s TruSeq small RNA sample preparation Kits (San Diego, CA, USA). The miRNA sequencing was performed by LC-BIO (Hangzhou, China). The ACGT101-miR program was used to eliminate junk, adapter dimers, common RNA families (snoRNA, snRNA, tRNA, rRNA), low complexity, and repeats from raw reads. Subsequently, unique sequences (18–25 nt in length) were aligned against in miRBase 21.0 (ftp://mirbase.org/pub/mirbase/ (accessed on 21 November 2018)) to identify known or derived miRNAs. Unmapped sequences were used to predict novel miRNAs using BLAST tools and RNAfold software (http://rna.tbi.univie.ac.at/cgi-bin/RNAWebSuite/RNAfold.cgi (accessed on 9 November 2018)). Normalized deep-sequencing counts represented the expression level of miRNA. MiRNAs with log_2_(fold change ratio) > 1 and *p*-value < 0.05 were considered as differentially expressed miRNAs (DEMs). miRNAs with a maximum expression level among all samples less than 10.00 were categorized as low-expression miRNAs, those with a maximum expression level higher than the average expression level of all miRNAs were considered as high-expression miRNAs, and the remaining miRNAs were categorized as middle-expression miRNAs.

### 4.4. Bioinformatics Analysis Tools

The RNA structure of the precursor of miRNA was predicted by Mfold [43]. psRNATarget was employed to identify targeted mRNA of DEMs [44]. The targeted genes were annotated on the National Rice Data Center website (http://www.ricedata.cn/gene (accessed on 8 April 2019)). Gene expression levels of *ARPS* were analyzed using our 84 RNA-seq datasets, as described in our previous study [33]. The Gene Ontology (GO) enriched terms of miRNA targets were analyzed by using AgriGO (https://systemsbiology.cau.edu.cn/agriGOv2/ (accessed on 14 October 2018)). Venn diagram analyses and a heatmap diagram were created by TBtools [45,46].

### 4.5. Quantitative Real-Time Polymerase Chain Reaction (qRT-PCR) Analysis

The total RNA samples were extracted as templates. The Transcroptor First Strand cDNA Synthesis Kit (Roche, Mannheim, Germany) was used for reverse transcription. The cDNA of targeted genes was typically reverse-transcribed following the manufacturer’s instructions of the kit. *Ubiquitin* was used as the internal control gene for qRT-PCR of candidate genes. For the reverse transcription of DEMs, miRNA-specific stem-loop RT primers replaced the OligodT20 primer in Transcroptor First Strand cDNA Synthesis Kit. The reaction was incubated at 16 °C for 30 min; then, pulsed RT of 60 cycles occurred at 30 °C for 30 s, 42 °C for 30 s, 50 °C for 1 s; 85 °C for 5 min to stop the reaction. *U6* snRNA was used as the internal control gene for qRT-PCR of DEMs. The qRT-PCRs were performed as described by Lu et al. [33]. Relative expression levels were calculated using the 2^−ΔΔCT^ method [47]. All primers were designed by using Primer Premier 5.0 software and the Primer-BLAST website (https://www.ncbi.nlm.nih.gov/tools/primer-blast/ (accessed on 12 December 2018)) (Table S8).

### 4.6. Dual-GFP Assay

A combined sequence, consisting of one eGFP, one CaMV poly A terminative sequence, one 35S promoter, one nuclear localization peptide, and another eGFP, was combined in *Hind*III and *Kpn*I cloning sites of a pGreenII 62SK vector to construct a Dual-GFP vector. The *35S::NLS-eGFP* in the Dual-GFP vector served as the internal reference signal. A fragment (946 to 1470 bp, Figure S8) from the *ARPS* coding sequence (*LOC_Os11g17290.3*) was PCR-amplified from H1 cDNA, and was combined into the Dual-GFP vector to construct the *ARPS-DGFP* vector. Subsequently, the mutant *ARPS* coding sequence (Figure S8) was obtained by overlapping PCR, and combined into the Dual-GFP vector to construct the *ARPSm-DGFP* vector. In addition, the sequence of *Ha1-MIR9564* (92 bp, Figure 7B) was combined with the *Ubiquitin* promoter to construct the *Ubi::MIR9564* vector. The *Ubi* control and *Ubi::MIR9564* were used as effecters, while *ARPS-DGFP* and *ARPSm-DGFP* were used as reporters. One effecter and one reporter were co-transformed into protoplasts extracted from a Taichung65 sheath, which were observed under a CLSM system. The related primers are listed in Table S8.

### 4.7. Over-Expression Line of ARPS in Neo-Tetraploid Rice

The coding sequence of *ARPS* (2883 bp) was PCR-amplified from H1 cDNA, and combined with the *Ubiquitin* promoter to construct *Ubi::ARPS*. The *Agrobacterium tumefaciens* EHA105 harboring above the constructed vector was transformed into Calli of H1 for transgenic line generation by BioRun (Wuhan, China). Transgenic seedlings and wild-type H1 were collectively assessed under the field condition at the experimental station of South China Agricultural University, Guangzhou, China.

## Figures and Tables

**Table 1:** Fertility comparison among H1, HF, and LF.

Materials	Seed Setting (%)	I_2_/KI-Stained Pollen Grains		TTC-Stained Pollen Grains	
		Normal (%)	Number	Viable (%)	Number
H1	74.38 ± 1.90 A	97.10 ± 0.26 A	2770	46.93 ± 4.26 A	5697
HF	71.39 ± 1.91 A	90.31 ± 1.58 A	3741	65.75 ± 2.78 A	6452
LF	5.17 ± 0.80 C	26.02 ± 8.06 B	3560	17.95 ± 6.27 B	3907

Note: TTC indicates 2,3,5-Triphenyl-2H-tetrazolium chloride solution. Number indicates total number of observed pollen grains. SEs were used here with *n* ≥ 3. Significant differences are indicated by different uppercase letters (one-way ANOVA, least significant difference (LSD) test, *p* < 0.01).

## Data Availability

Data are contained within the article and Supplementary Materials.

## Supplementary Materials

The following supporting information can be downloaded at: https://www.mdpi.com/article/10.3390/plants13111461/s1, Figure S1: The breeding procedure of high-fertility (HF) and low-fertility (LF) populations. Figure S2: Plant phenotypes of parents and F_1_ hybrids of LF × HF and LF × H1. Figure S3: Mature pollen and other abnormalities during double fertilization. Figure S4: Pearson correlation of miRNA-seq among biological replicates of each library. Figure S5: Gene Ontology (GO) enrichment analyses of predicted targets of coDEMs. Figure S6: Subcellular localization of Dual-GFP signal with NLS-mCherry. Figure S7: Pre-experiment of pollen grains stained with 1% TTC solution. Figure S8: Coding sequence fragment of ARPS and ARPSm used for Dual-GFP assay. Table S1: miRNA-seq data quality in H1, HF, and LF. Table S2: Information of miRNA detected from H1, HF, and LF. Table S3: Differentially expressed miRNAs (DEMs) in meiotic anthers between H1 and LF. Table S4: Differentially expressed miRNAs (DEMs) in meiotic anthers between HF and LF. Table S5: Common differentially expressed miRNAs (coDEMs) in meiotic anthers between H1-HF and LF. Table S6: Common differentially expressed genes (coDEGs) in meiotic anthers between H1-HF and LF [33]. Table S7: CoDEMs–coDEGs in meiotic anthers between H1-HF and LF. Table S8: Primers’ information.
