# Supplementary material for: Cytological Observation and RNA-Seq Analyses Reveal miR9564 and Its Target Associated with Pollen Sterility in Autotetraploid Rice
Source: Plants (Basel). 2024 May 24;13(11):1461. doi: 10.3390/plants13111461 (PMC11175005; doi:10.3390/plants13111461)
Supplement: Supplementary file 1 [file plants-13-01461-s001.zip › plants-2979190-supplementary Figures.pdf]

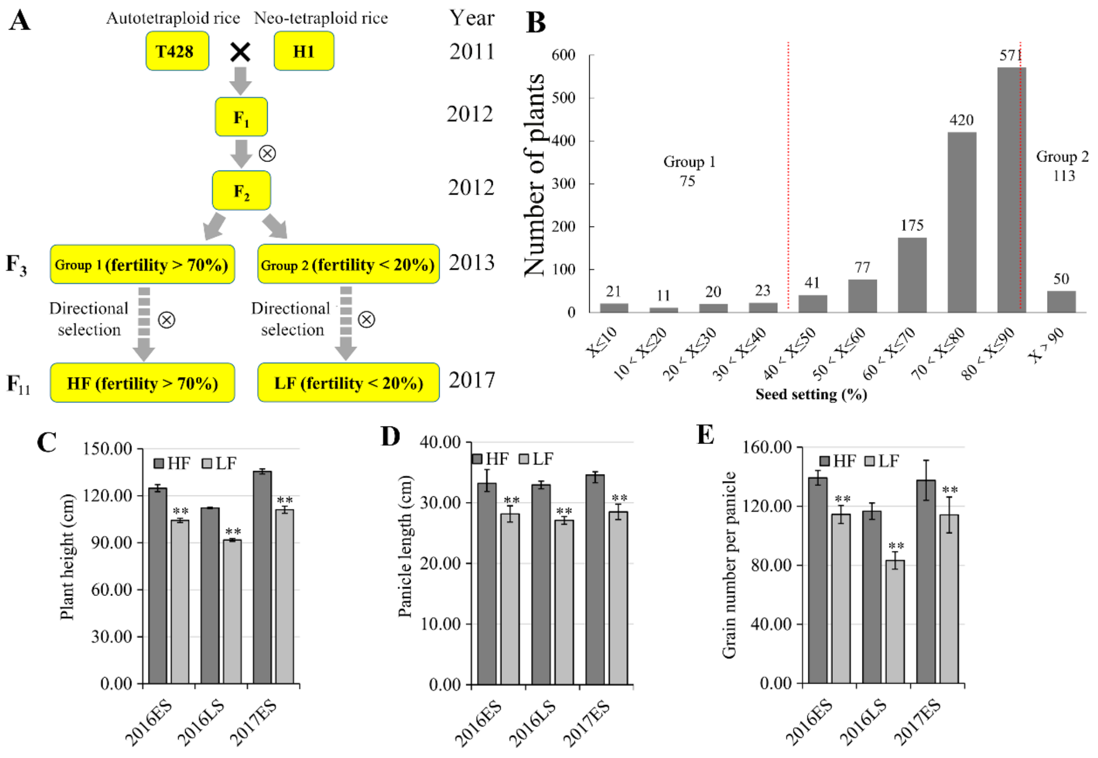

Figure S1 The breeding procedure of high fertility (HF) and low fertility (LF) populations

Note: (A) The detailed breeding procedure. (B) The distribution of seed setting of F_2 population (T428×H1). Red dotted lines in figure indicate demarcation of group 1 or group 2. (C-E) Plant height (C), panicle length (D), grain number per panicle (E) of HF and LF. ES indicates early season. LS indicates late season. Error bars indicate the SE with n ≥ 3. ** $P < 0.01$, Student's *t*-test.

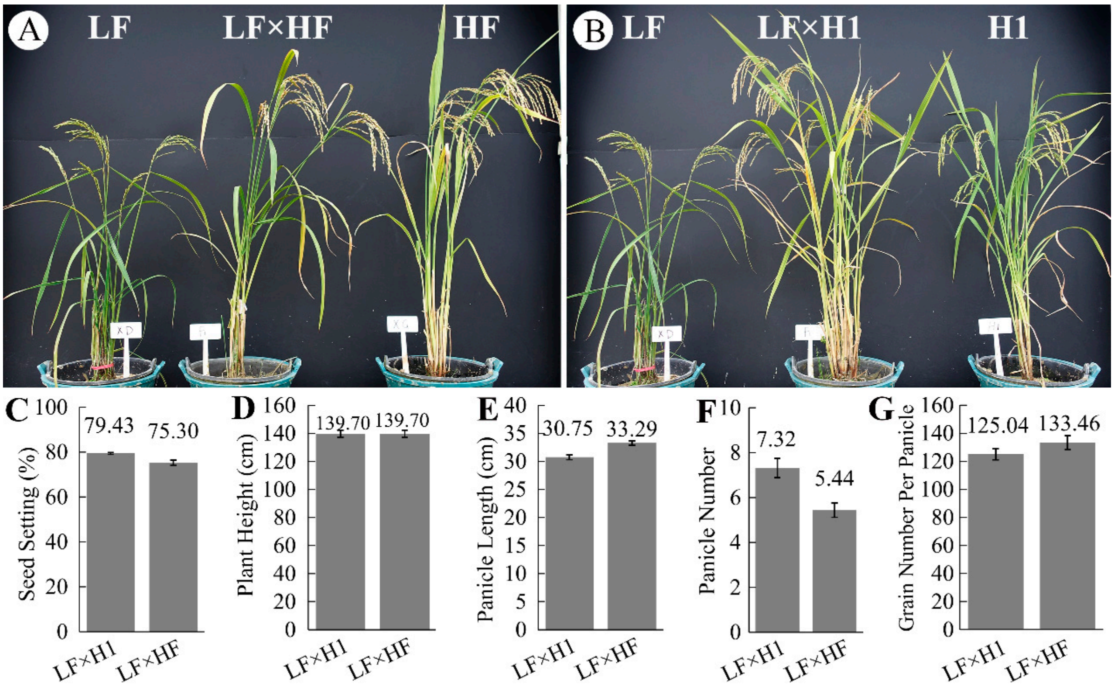

Figure S2 Plant phenotypes of parents and F_1 hybrids of LF×HF and LF×H1

(A) F_1 hybrid of LF×HF. (B) F_1 hybrid of LF×H1. (C-G) Main agronomic traits of F_1(LF×H1) and

F_1(LF×HF). (C) Seed setting, (D) plant height, (E) panicle length, (F) panicle number, (G) grain number per panicle. The error bars indicate the ±SE. H1/HF and LF represent the high and low fertility tetraploid rice, respectively.

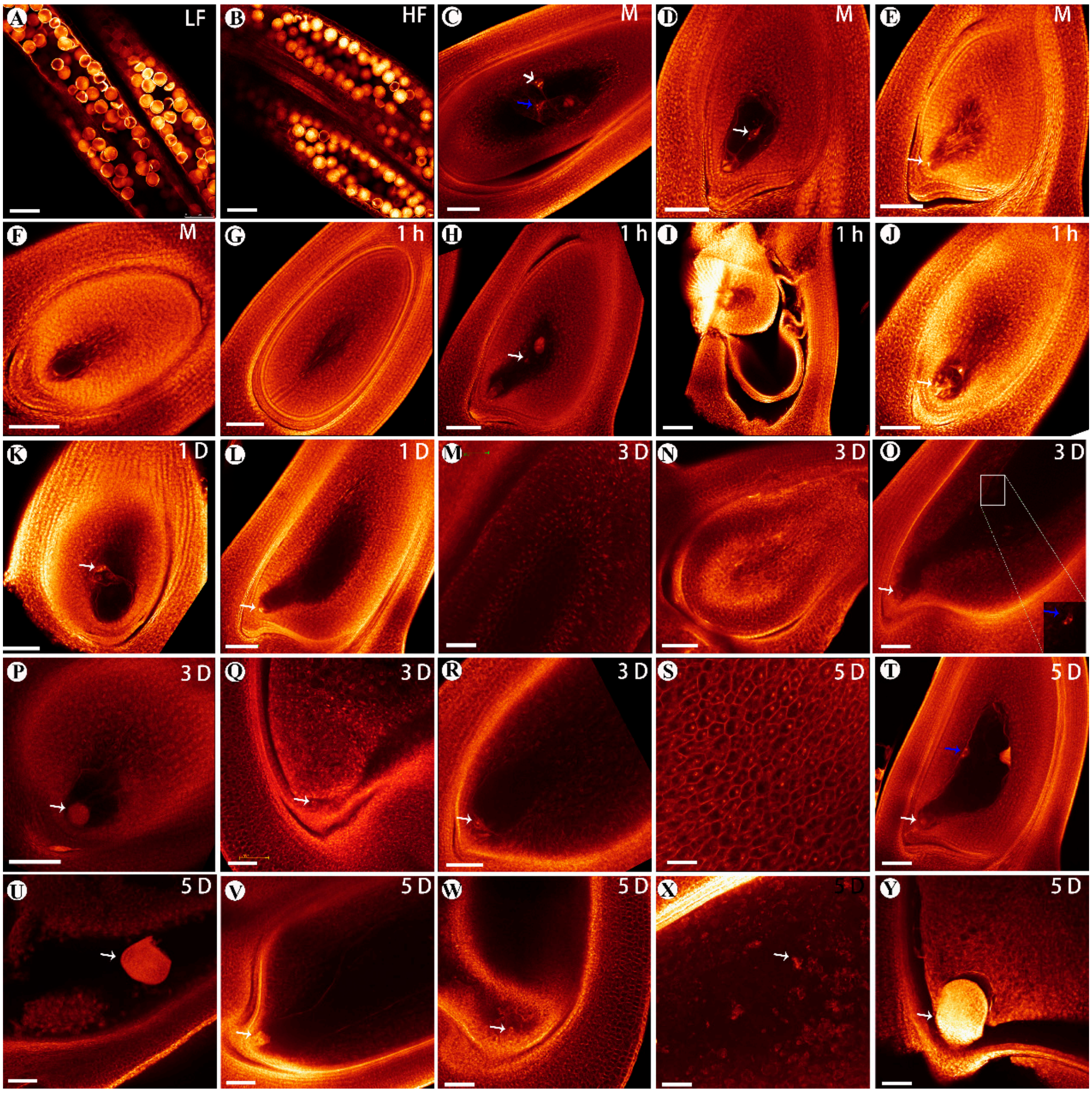

Figure S3 Mature pollen and other abnormalities during double fertilization.

(A-B) Mature pollen of LF and HF. (C-D) Embryo sac with abnormal number and atypical position of polar nuclei (arrows). (E-G) Degraded embryo sac. The arrow indicates the degraded egg apparatus. (H) Unfertilized embryo sac with atypical positioning polar nuclei. The arrows indicate the polar nuclei. (I) Undeveloped embryo sac. (J) Poly eggs (arrow). (K) Unfertilized ovule with multiple polar nuclei (arrow). (L) Degraded zygote (arrow). (M) Normal endosperm. (N) Degraded embryo sac. (O) Inflated but unfertilized ovule with egg cell (arrow) and atypical positioning polar (blue arrow). (P) Single fertilized embryo. (Q) Single fertilized endosperm with unfertilized egg cell (arrow). (R) Inflated but unfertilized ovule. Arrow indicates the egg cell. (S) Normal endosperm. (T) Inflated but unfertilized ovule with egg cell (arrow) and atypical positioning polar nuclei (blue arrow). (U) Embryo at abnormal position (arrow). (V-W) Inflated ovule with unfertilized egg cell (arrow). (X) Abnormal hyperplasia (arrow) in nucellar cells of unfertilized ovule. (Y) Retardative development of embryo. M, mature; 1 h,

1 D, 3 D, 5 D, samples at 1 hour, 1 day, 3 days, 5 days after-flowering, respectively. Bars = 100 μm

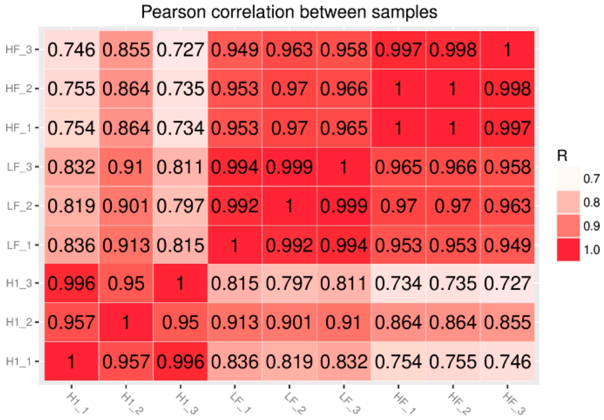

Figure S4 Pearson correlation of miRNA-seq among biological replicates of each library.

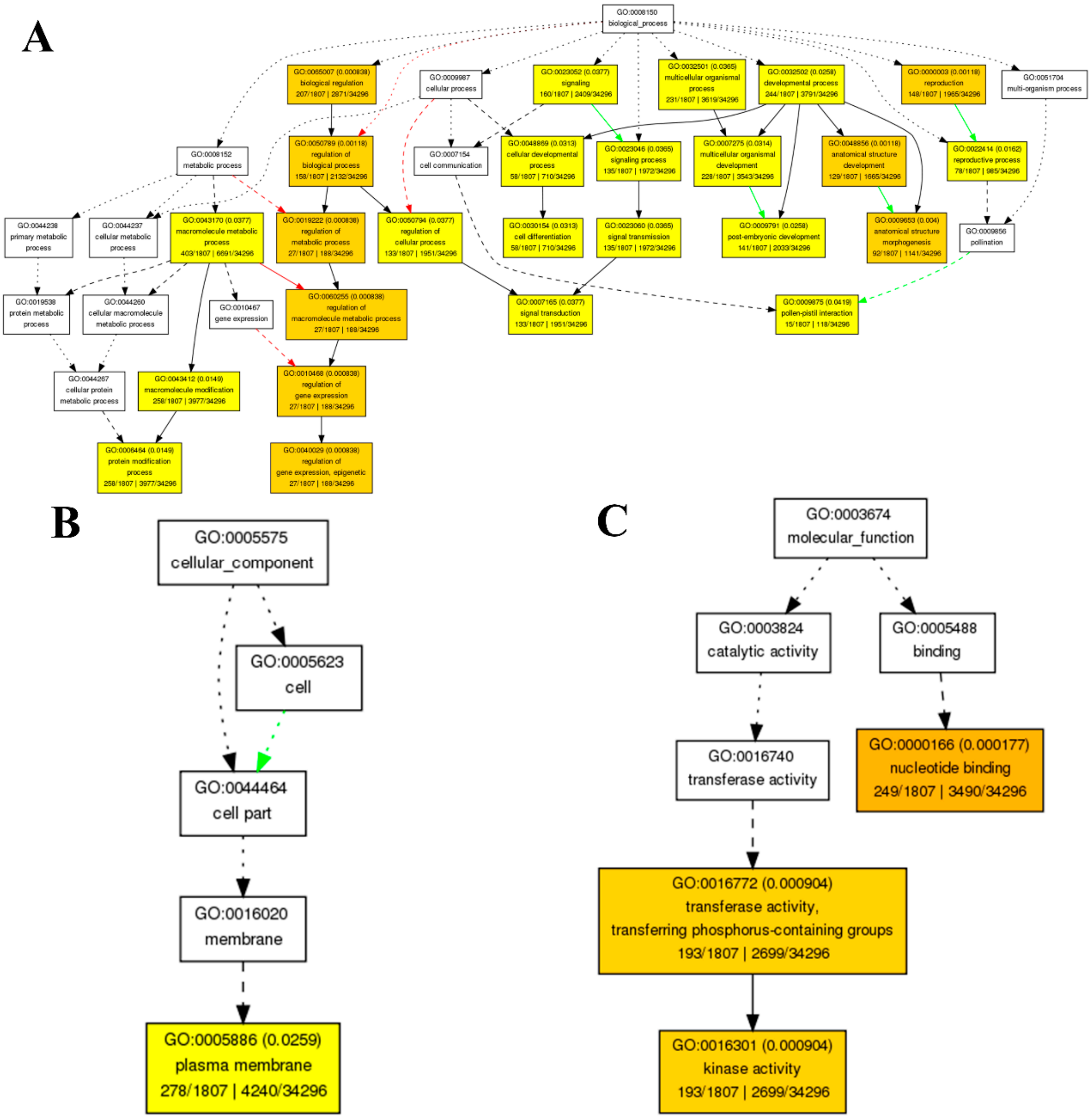

Figure S5 Gene Ontology (GO) enrichment analyses of predicted targets of coDEMs.

(A) Biological process items; (B) Cellular component items; (C) Molecular function items. coDEMs, common differentially expressed miRNAs in both H1/LF and HF/LF.

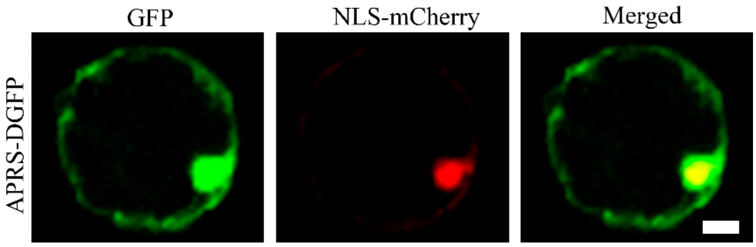

Figure S6. Subcellular localization of Dual-GFP signal with NLS-mCherry.

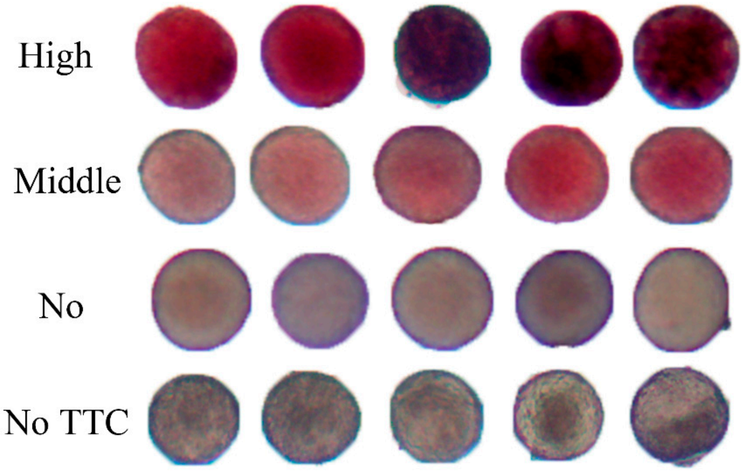

Figure S7 Pre-experiment of pollen grains stained with 1% TTC solution.

High indicated those pollen grains stained as deep red. Middle indicated those pollen grains stained as light red. Both High- and Middle-type pollen grains were identified as viable pollen grains. “No” showed devitalized pollen grains. “No TTC” showed pollen grains only treated by PBS solution.

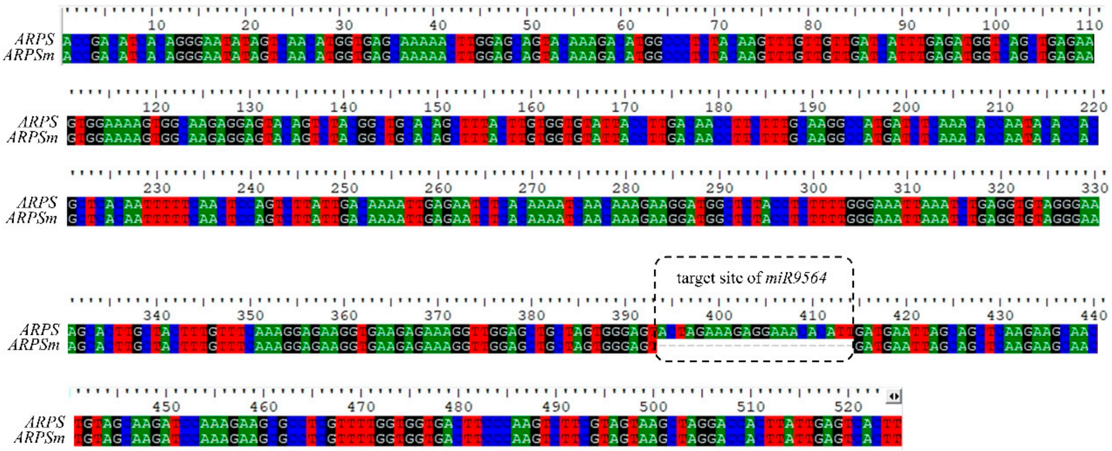

Figure S8 Coding sequence fragment of *ARPS* and *ARPSm* using for Dual-GFP assay
